# A Genome-Wide Screen Reveals That Endocytic Genes Are Important for Pma1p Asymmetry during Cell Division in *Saccharomyces cerevisiae*

**DOI:** 10.3390/ijms23042364

**Published:** 2022-02-21

**Authors:** So-Young Yoon, Eunhong Jang, Naho Ko, Minseok Kim, Su Yoon Kim, Yeojin Moon, Jeong-Seok Nam, Sunjae Lee, Youngsoo Jun

**Affiliations:** 1 School of Life Sciences, Gwangju Institute of Science and Technology, 123 Cheomdangwagi-ro, Buk-gu, Gwangju 61005, Korea; sparrow0214@gist.ac.kr (S.-Y.Y.); eunhong0714@gist.ac.kr (E.J.); konaho4048@gist.ac.kr (N.K.); shs161107@gist.ac.kr (M.K.); suyoonkim@gist.ac.kr (S.Y.K.); yeojinmoon@gist.ac.kr (Y.M.); namje@gist.ac.kr (J.-S.N.); leesunjae@gist.ac.kr (S.L.); 2 Cell Logistics Research Center, Gwangju Institute of Science and Technology, 123 Cheomdangwagi-ro, Buk-gu, Gwangju 61005, Korea

**Keywords:** replicative lifespan, Pma1p, pH asymmetry, cellular aging, endocytosis, *Saccharomyces cerevisiae*

## Abstract

An asymmetry in cytosolic pH between mother and daughter cells was reported to underlie cellular aging in the budding yeast *Saccharomyces cerevisiae*; however, the underlying mechanism remains unknown. Preferential accumulation of Pma1p, which pumps cytoplasmic protons out of cells, at the plasma membrane of mother cells, but not of their newly-formed daughter cells, is believed to be responsible for the pH increase in mother cells by reducing the level of cytoplasmic protons. This, in turn, decreases the acidity of vacuoles, which is well correlated with aging of yeast cells. In this study, to identify genes that regulate the preferential accumulation of Pma1p in mother cells, we performed a genome-wide screen using a collection of single gene deletion yeast strains. A subset of genes involved in the endocytic pathway, such as *VPS8*, *VPS9*, and *VPS21*, was important for Pma1p accumulation. Unexpectedly, however, there was little correlation between deletion of each of these genes and the replicative lifespan of yeast, suggesting that Pma1p accumulation in mother cells is not the key determinant that underlies aging of mother cells.

## 1. Introduction

Cellular aging is a fundamental process that contributes to the complicated aging process at the organismal level [1]. Cellular aging can be considered from two perspectives [2]. First, the replicative lifespan (RLS) represents how many times cells divide before they stop dividing and subsequently die. This has been useful to examine aging of cells that repeatedly divide, such as epithelial cells. Second, the chronological lifespan (CLS) represents how long non-dividing cells live before they die. Cellular aging of neurons, which almost never divide after they have completed differentiation, has been analyzed using CLS. Replicative aging in the budding yeast *Saccharomyces cerevisiae* is defined as the number of times an individual cell divides [3]. During replicative aging, mother cells produce a limited number of daughter cells before division stops. This replicative aging process is asymmetric: while mother cells age, their daughter cells are rejuvenated. It has been proposed that several asymmetric phenotypes [4,5,6], such as mitochondrial fragmentation and an increased level of mitochondrial reactive oxygen species, contribute to aging of mother cells. Another proposed determinant of mother cell-specific aging is asymmetry in cytoplasmic pH between mother and daughter cells [7,8]. As mother cells age, their vacuolar pH increases, which inactivates a variety of acid hydrolases in vacuoles. This increase in vacuolar pH decreases the mitochondrial membrane potential in mother cells by unknown mechanisms [8]. Thus, these dual defects in vacuoles and mitochondria may contribute to aging of mother cells. By contrast, daughter cells are generated largely free of aging factors after every division. This phenomenon is termed rejuvenation of daughter cells and is partly achieved by retention of damaged mitochondria [9] and the plasma membrane proton pump Pma1p [7], preferentially in mother cells. Pma1p is the major proton pump at the plasma membrane in budding yeast and transports cytosolic protons to the exterior of cells at the expense of ATP hydrolysis [10]. Because Pma1p is a plasma membrane protein, it was expected to be evenly distributed between mother and daughter cells during cell divisions, but it was found to preferentially accumulate in mother cells [7]. Pma1p is also a long-lived protein that survives throughout the lifespan of yeast cells (25–30 divisions). Thayer et al. isolated ~135 proteins that were designated long-lived asymmetrically retained proteins (LARPs), one of which was Pma1p [11]. According to studies performed by Gottschling and colleagues [7,8,11], Pma1p preferentially accumulates at the plasma membrane of mother cells during cell divisions, and the resulting decreased concentration of cytoplasmic protons leads to a scarcity of vacuolar protons, which eventually increases vacuolar pH. Despite the importance of the preferential accumulation of Pma1p in mother cells, the underlying molecular mechanism is completely unknown. In this study, using a collection of single gene deletion yeast strains, we performed a genome-wide screen for genes that regulate the preferential accumulation of Pma1p at the plasma membrane of mother cells and identified a series of genes involved in the endocytic pathway as candidate genes that may regulate aging of yeast cells.

## 2. Results

### 2.1. A Genome-Wide Screen to Identify Genes That Regulate the Asymmetric Distribution of Pma1p between Mother and Daughter Cells during Cell Division

**Figure 1:**
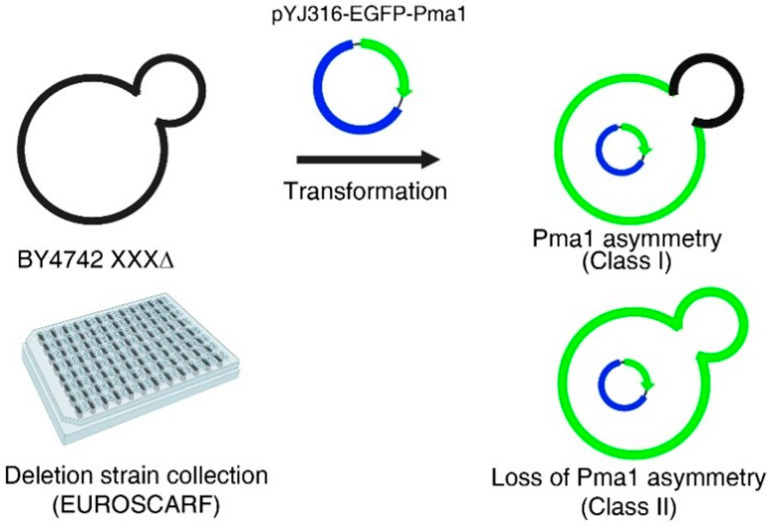
Schematic representation of the genome-wide screen identifying genes that regulate Pma1p asymmetry during cell division. Yeast cells from a collection of single-gene deletion mutants (EUROSCARF, Scientific Research and Development GmbH, Germany) were transformed with a plasmid DNA encoding Pma1p-EGFP, and GFP fluorescence along the plasma membrane of mother and daughter cells was analyzed by fluorescence microscopy.

During cell division, the plasma membrane protein Pma1p asymmetrically accumulates in the mother cell, instead of being evenly distributed between mother and daughter cells, in the budding yeast *Saccharomyces cerevisiae* [7]. This asymmetric distribution of Pma1p has been suggested to contribute to aging of mother cells and rejuvenation of daughter cells because Pma1p accumulation leads to depletion of cytoplasmic protons, which, in turn, increases vacuolar pH, a hallmark of cellular aging in yeast [8]. To identify genes that regulate the asymmetric distribution of Pma1p during cell division, we performed a genome-wide screen using a collection of single gene deletion yeast strains. To this end, we transformed yeast cells with a plasmid encoding EGFP-fused Pma1p under the control of the *ADH1* promoter (*pADH1*), which is a constitutively strong promoter, and analyzed Pma1p-EGFP under a fluorescence microscope (Figure 1). About 30% of wild-type cells were classified as the Class II phenotype (symmetric distribution of Pma1p). We sought to identify mutants in which more than 50% of cells had the Class II phenotype. Out of about 5000 single-gene deletion mutants, 12 mutants were finally isolated (Table 1).

Interestingly, the 12 mutants were all defective for genes involved in endocytosis. Among these, we focused on three genes, *VPS8*, *VPS9*, and *VPS21*, in the current study because deletion of these genes resulted in the highest percentages of cells with the Class II phenotype. More interestingly, these three genes all encode proteins involved in CORVET-mediated stages during endocytosis [12,13,14,15,16,17,18]. Cells lacking any of these genes can endocytose plasma membrane proteins, such as Pma1p, but are defective in their sorting to vacuoles. Recycling from early endosomes to the plasma membrane is still possible, partly explaining why deletion of any of these genes results in further accumulation of Pma1p at the plasma membrane and possibly leads to leakage of Pma1p into the plasma membrane of daughter cells.

**Figure 2:**
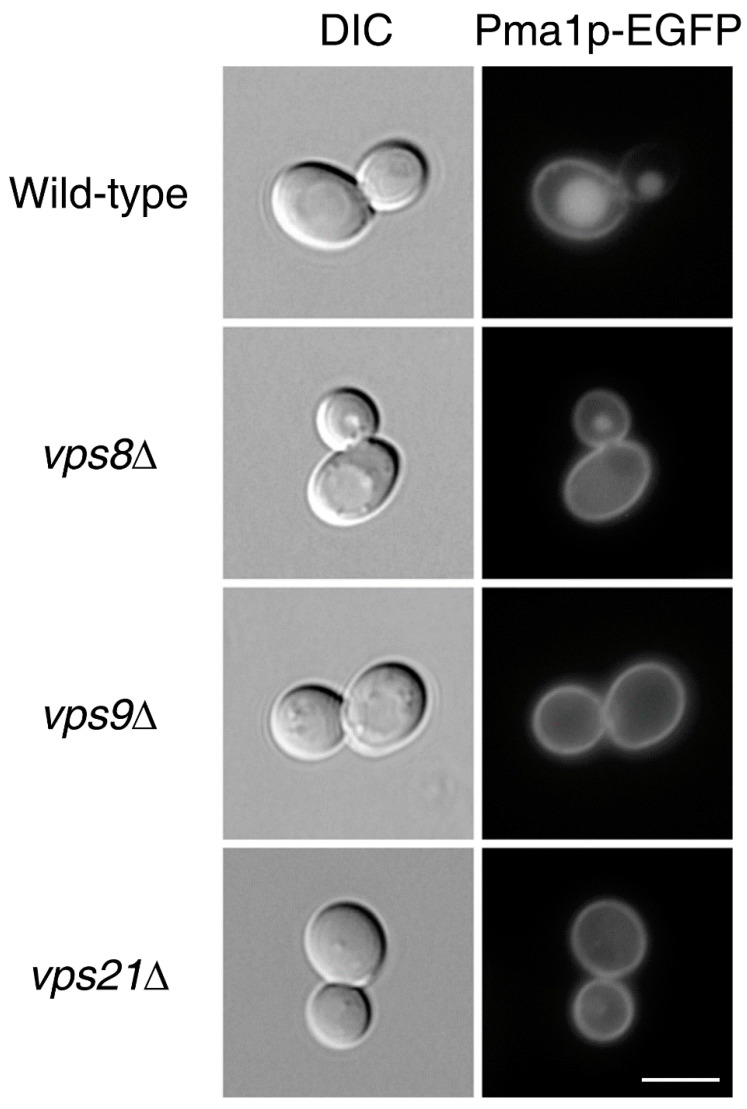
Deletion of *VPS8*, *VPS9*, or *VPS21* disrupts Pma1p-EGFP asymmetry between mother and daughter cells during cell division. Wild-type cells or cells deleted for *VPS8*, *VPS9*, or *VPS21* expressing Pma1p-EGFP under the *PMA1* promoter were analyzed by fluorescence microscopy (magnification: 100×). Differential interference contrast (DIC) images (**left**) and GFP fluorescence images (**right**) are shown. About 100 cells per wild-type or mutant were analyzed, and representative images are shown. Scale bar: 5 μm.

For our genome-wide screen, we over-expressed Pma1p-EGFP in yeast cells bearing endogenous Pma1p. Thus, it is possible that over-expressed Pma1p artificially resulted in the increased percentage of cells with the Class II phenotype. To exclude this possibility, we attempted to fuse the gene encoding EGFP to the *PMA1* gene in the chromosome. In this way, we were able to express Pma1p-EGFP in the absence of untagged, endogenous Pma1p. Pma1p-EGFP was evenly distributed between mother and daughter cells in cells lacking *VPS8*, *VPS9*, or *VPS21*, regardless of whether Pma1p-EGFP was encoded in an episomal plasmid or in the chromosome (Figure 2).

### 2.2. Endocytic Genes Seem to Be Critical for the Asymmetric Distribution of Plasma Membrane LARPs but Not of Cytoplasmic LARPs

**Figure 3:**
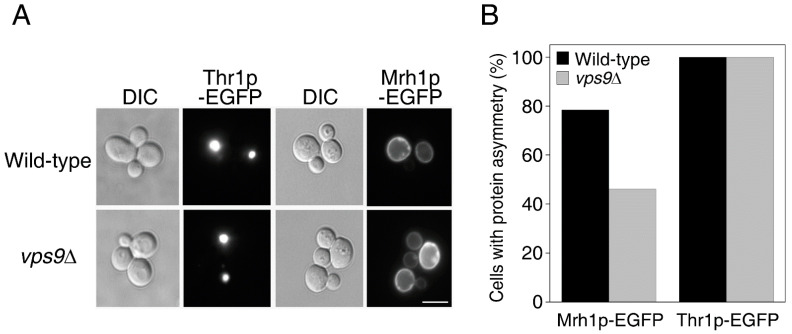
Deletion of *VPS9* disrupts the asymmetry of Mrh1p, a plasma membrane protein, but that of Thr1p, a cytoplasmic protein. Wild-type or *vps9*Δ cells were transformed with plasmid DNA encoding Thr1p-EGFP or Mrh1p-EGFP and analyzed for EGFP fluorescence by fluorescence microscopy. More than 100 cells were analyzed, and representative images (**A**) and their quantitation (**B**) are shown. Scale bar: 5 μm.

Pma1p is a member of the LARP family [11]. LARPs are defined as proteins that are retained within mother cells as they undergo repeated asymmetric divisions and that generally contribute to the age-associated phenotype of mother cells [11]. To test whether genes that regulate the asymmetric distribution of Pma1p are also involved in the asymmetric distribution of other LARPs or are specifically involved in that of Pma1p, we expressed EGFP-fused Mrh1p, a plasma membrane LARP, or Thr1p, a cytoplasmic LARP that forms short rod-like structure [11,19], in *vps9*Δ cells. While Thr1p-EGFP was asymmetrically distributed in both wild-type and *vps9*Δ cells (Figure 3, left), the asymmetric distribution of Mrh1p-EGFP was suppressed by deletion of *VPS9* (Figure 3, right). These results indicate that genes that regulate the asymmetric distribution of Pma1p also play a role in the asymmetric distribution of other plasma membrane LARPs during cell division.

### 2.3. Disruption of the Asymmetric Distribution of Pma1p during Cell Division Correlates Little with RLS

**Figure 4:**
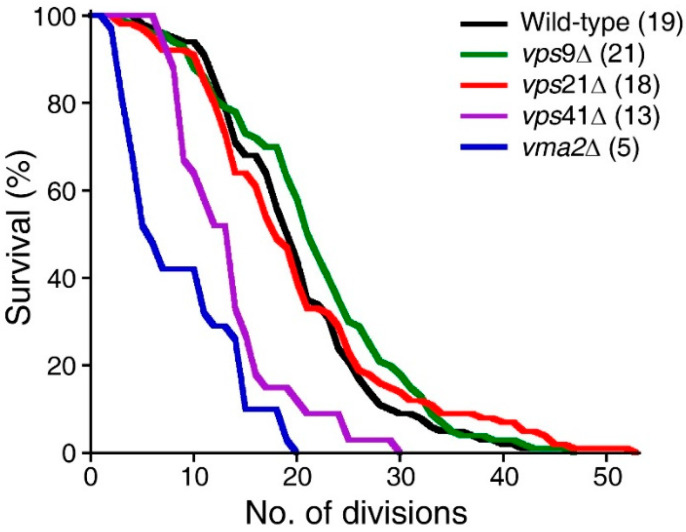
Pma1p asymmetry little correlates with the RLS of the budding yeast *Saccharomyces cerevisiae*. The RLS of wild-type, *vps9*Δ, *vps21*Δ, *vps41*Δ, and *vma2*Δ cells was measured by micromanipulation as described in the Materials and Methods section. Median lifespan is indicated in parenthesis (*n* = 32 for wild-type, *n* = 32 for *vps9*Δ, *n* = 32 for *vps21*Δ, *n* = 33 for *vps41*Δ, and *n* = 31 for *vma2*Δ).

Retention of Pma1p in mother cells during cell division and its resulting accumulation in mother cells over repeated cell divisions were suggested to be one of the major factors that affect the RLS of the budding yeast *Saccharomyces cerevisiae* [7]. Pma1p activity antagonizes vacuole acidification: over-expression of Pma1p drastically increases vacuolar pH, whereas yeast cells bearing the *pma1-105* allele, which encodes Pma1p with reduced activity, retain vacuolar acidity after at least 18 divisions [7]. Consistent with the idea that the level of Pma1p at the plasma membrane correlates well with RLS, the RLS of cells bearing the *pma1-105* allele is about 30% higher than that of wild-type cells [7]. To examine whether genes that regulate the asymmetric distribution of Pma1p affect RLS, the RLS of wild-type, *vma2*Δ, *vps9*Δ, *vps21*Δ, and *vps41*Δ cells was measured. Yeast cells lacking the V-ATPase (vacuolar proton pump) subunit Vma2p had a short median lifespan of five divisions (Figure 4, blue line), as previously reported [7]. The median lifespan of wild-type cells was 19 divisions. Interestingly, however, the median lifespans of *vps9*Δ and *vps21*Δ cells were 21 and 18 divisions, respectively, which were comparable with that of wild-type cells. Interestingly, *vps41*Δ cells had a relatively short median lifespan of 13 divisions (Figure 4, purple line), although only about 56% of these cells had the Class II phenotype, which was markedly lower than the corresponding percentages of *vps9*Δ and *vps21*Δ cells (see Table 1). These results suggest that there is no clear correlation between Pma1p accumulation and RLS, and loss of accumulation of Pma1p in mother cells, *per se*, is not sufficient to affect their RLS.

### 2.4. Damaged Vacuoles Are Successfully Transported to Daughter Cells during Cell Division

**Figure 5:**
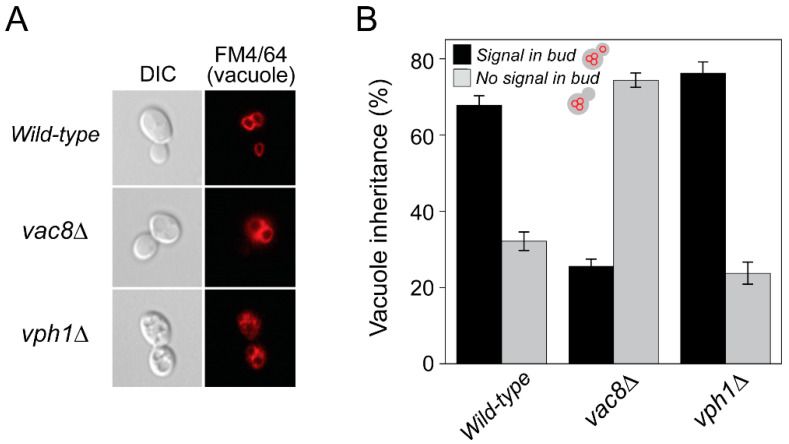
Damaged yeast vacuoles are normally inherited by daughter cells during cell division. Wild-type or mutant yeast cells were grown in the presence of FM4/64, a lipophilic dye for vacuoles, and analyzed for vacuole inheritance during cell division. Yeast cells lacking Vac8p, a vacuolar protein essential for vacuole inheritance, were used as control. Representative images from each cell type (**A**) and quantitation for vacuole inheritance (**B**) are shown. Magnification: 100×. DIC, differential interference contrast.

It was reported that an increase in vacuolar pH at an early age limits the lifespan of yeast and mother-daughter asymmetry of Pma1p and that vacuolar pH thereby underlies aging of mother cells [7,8]. Segregation of damaged mitochondria to daughter cells is blocked during mitosis and, thus, provides daughter cells with healthy mitochondria, which contributes to aging of mother cells and rejuvenation of daughter cells [4,20,21]. Therefore, we examined whether damaged vacuoles (with an increased pH) are evenly segregated into daughter cells during cell division. To this end, wild-type or mutant cells (*vac8*Δ and *vph1*Δ) were labeled with FM4/64, a lipophilic dye that eventually accumulates in the vacuolar membrane of live yeast cells [22], and vacuoles in mother and daughter cells were analyzed by fluorescence microscopy. Because Vac8p is required for vacuole inheritance [23,24], significant portions of emerging buds of *vac8*Δ cells failed to acquire vacuoles from mother cells. Yeast cells lacking Vph1p, a subunit of the vacuole ATPase responsible for vacuolar acidification [25], had no defect in vacuole segregation compared with wild-type cells (Figure 5). These data suggest that, unlike damaged mitochondria, damaged vacuoles are normally transported to daughter cells, excluding the possibility that asymmetric segregation of damaged vacuoles underlies aging of mother cells.

## 3. Discussion

In this study, by performing a genome-wide screen, we identified a series of candidate genes that may regulate the preferential accumulation of Pma1p at the plasma membrane of mother cells. Interestingly, most of these genes are involved in endocytosis. One simplistic explanation of this screening result is that defects in endocytosis block endo-lysosomal degradation of Pma1p, which, in turn, considerably increases the level of Pma1p at the plasma membrane of mother cells and allows diffusion of a portion of excess Pma1p to the plasma membrane of daughter cells. However, because only a small proportion of endocytic genes were identified, general defects in endocytosis are unlikely sufficient to disrupt Pma1p asymmetry between mother and daughter cells. Furthermore, we observed the strongest phenotypes in yeast cells deficient for one of three genes, *VPS8*, *VPS9*, and *VPS21*, which are involved in the same pathway during endocytosis [17,18]. Vps21p is a Rab GTPase required for transport from endosomes to vacuoles. It is functional only when bound to GTP. This GTP binding is mediated by Vps9p, which exchanges GDP bound to Vps21p with GTP, thereby activating Vps21p. Vps8p is a component of the CORVET complex, a six-subunit complex required for transport from late endosomes to vacuoles, and binds only to the GTP-bound form of Vps21p. Thus, Vps8p is an effector of activated Vps21p [17]. Is the CORVET complex, or its function, critical for Pma1p asymmetry? Although another CORVET component, Vps3p, was one of our identified candidates, deletion of any of the other four components (Vps11p, Vps16p, Vps18p, and Vps33p) did not markedly disrupt Pma1p asymmetry, suggesting that a CORVET-independent function of Vps8p/Vps9p/Vps21p mediates Pma1p accumulation in mother cells. It was also reported that Pma1p delivery to the plasma membrane requires its association with lipid rafts [26]. Thus, the lipid rafts of mother cells may be different from those of daughter cells, which may, in turn, affect the asymmetry of Pma1p between mother and daughter cells. Interestingly, however, the effect of lipid rafts on the asymmetric distribution of plasma membrane proteins during cell divisions remains entirely uncharacterized. Thus, future studies will be required to examine this intriguing issue.

**Figure 6:**
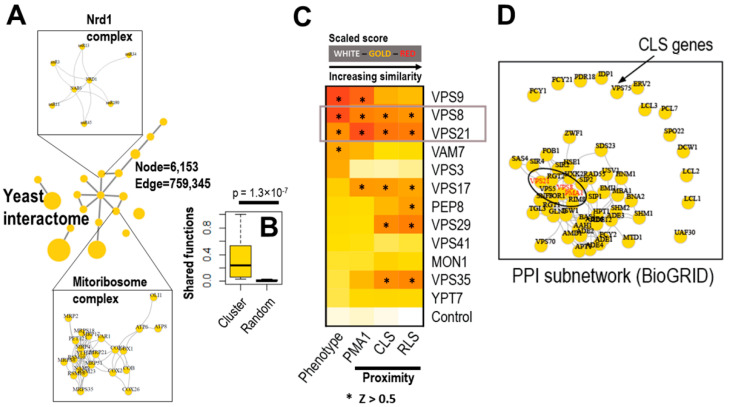
A systems biology approach reveals that Vps8p and Vps21p interact with various proteins involved in CLS and RLS. The systems biology approach identified key drivers of the aging process, such as *VPS8* and *VPS21*. (**A**) Yeast protein interaction networks are generated from the BioGRID database (6153 nodes and 759,345 edges). This shows protein clusters of the protein interaction networks (nodes are protein clusters, and edges are significant interactions between clusters). The top box indicates protein interactions of the corresponding clusters, which are enriched with the Nrd1 complex proteins. The bottom box shows protein interactions of the corresponding clusters, enriched with the mitoribosome complex proteins. (**B**) Proteins in clusters share molecular functions (i.e., GO functions), significantly more than randomly-selected proteins (Wilcoxon test *p*-value = 1.3 × 10^−7^). (**C**) Based on network proximity measures, the 12 screened genes (rows of the heatmap) are significantly connected with *PMA1* (second column), CLS (third column), and RLS genes (fourth column). Based on a scaled score (Z-score > 0.5), *VPS8* and *VPS21* are significantly higher on all occasions, likely being critical players in the aging process. The leftmost columns in the heatmap indicate phenotype changes in the 12 screened genes. (**D**) *VPS8* and *VPS21* are centrally located at the CLS interaction network, thereby governing the interactions of CLS genes.

The preferential accumulation of Pma1p in mother cells, at least in part, underlies aging of mother cells and rejuvenation of daughter cells. Therefore, we initially anticipated that disruption of Pma1p asymmetry would increase the RLS of mother cells by sharing an aging factor with their daughter cells. Unexpectedly, however, the RLS of yeast cells deficient for *VPS9* or *VPS21* was comparable with that of wild-type cells (Figure 4). Even more strikingly, yeast cells deficient for *VPS41* had a markedly decreased RLS, while deletion of *VPS41* caused a relatively mild defect in Pma1p asymmetry. Among genes identified in our screen, *YPT7*, *VAM7*, and *VPS41* were suggested to regulate yeast aging by modulating the function of vacuoles [27,28,29] or affecting a life-span extending form of autophagy [30]. Collectively, these results suggest that Pma1p asymmetry is not a major determinant that underlies aging of mother cells. Endocytosis influences many other cellular processes, and cellular aging is regulated by a variety of cellular processes. Therefore, loss-of-function studies using single-gene deletion mutants as described here may be inadequate to identify genes relevant to cellular aging and to analyze the underlying mechanisms. Thus, we employed a systems biology approach and uncovered *VPS8* and *VPS21* as key regulators of the cellular aging process (Figure 6). Cellular aging is a multifactorial process controlled by complex molecular and biochemical interactions. Such inherent complexity may necessitate systems biology approaches to identify “key drivers” that are hidden in the complex network. To this end, we first collected large-scale protein interactome data from the BioGRID database [31] and selected interactions originating from *Saccharomyces cerevisiae*. These totaled 759,345 interactions of 6153 yeast proteins (Figure 6A). Based on connectivity, we identified protein interaction clusters from the network (Figure 6A). Interestingly, we found many protein complexes that mediate critical cellular processes from the protein interaction clusters. For example, the mitochondrial ribosome complex (Figure 6A, bottom box) [32,33] and the Nrd1 complex that functions in transcription termination of non-coding RNAs (Figure 6A, top box) [34] were identified. Furthermore, we checked the functional similarity of proteins in the protein clusters and found that they have commonly-shared functions compared with randomly-selected proteins (Figure 6B). Therefore, essential key regulators can be found using our quantitative in silico approaches based on protein interaction connectivity. Next, based on this established network, we investigated if the 12 identified genes (see Table 1) closely interacted with Pma1p, a key regulator of the aging process, such as genes associated with CLS [28] and RLS [35], in previous studies (see the Materials and Methods Section 4). Based on network proximity measurement (see the Materials and Methods Section 4), we identified the network proximity of the 12 identified genes and found that many genes had high scores, such as *VPS9*, *VPS8*, *VPS21*, and *VAM7* (Z > 0.5) (Figure 6C). We also checked their proximity to other key genes essential for the aging process, such as CLS [28] and RLS [35] genes. Interestingly, we identified higher scores for *VPS8* and *VPS21* with CLS and RLS genes (Z > 0.5). Therefore, it is tempting to speculate that Vps8p and Vps21p function as key regulators by interacting with Pma1p and CLS/RLS-related proteins, thereby orchestrating the bio-molecular interactions involved in the aging process. We further delved into the interactome of Vps8p and Vps21p with CLS proteins and found that Vps8p and Vps21p, together with Pma1p, were key hubs of the CLS protein interactome (eclipse in Figure 6D).

## 4. Materials and Methods

### 4.1. Yeast Strains and Plasmids

All yeast strains used in this study were derived from BY4742 (*MATα his3*Δ *leu2*Δ *lys2*Δ *ura3*Δ). A collection of single gene knock-out yeast strains was purchased from EUROSCARF. The plasmid pYJ316-PMA1-EGFP was generated by inserting a PCR-amplified DNA fragment encoding PMA1-EGFP into pYJ316 [36]. The plasmid pYM28 for chromosomal tagging of the EGFP gene was purchased from EUROSCARF. The plasmids pYJ316-MRH1-EGFP and pYJ406-THR1-EGFP were generated by inserting PCR-amplified DNA fragments encoding MRH1-EGFP and THR1-EGFP into pYJ316 and pYJ406 [36], respectively.

### 4.2. Yeast Transformation

Yeast cells retrieved from a collection of single gene knock-out yeast strains (EUROSCARF) were grown on YPD (10 g/L yeast extract, 20 g/L peptone, and 20 g/L dextrose) at 30 °C for 2–3 days. Three or more healthy colonies were inoculated into each well of a 96-well round bottom plate containing YPDA (190 μL per well) and incubated at 30 °C overnight. Then, the plate received fresh YPDA (190 μL) into each well and was incubated at 30 °C. After 90 min, the plate was centrifuged (3000× g) at 25 °C for 5 min. The resulting supernatant was removed, and the pellet was resuspended in 12 μL of Buffer A (62.5 mM Tris-HCl, pH 8.0, 0.5 M EDTA, and 10 mg/mL boiled salmon sperm DNA) containing plasmids. After adding 80 μL of Buffer B (50% PEG3350 and 100 mM lithium acetate), the plate was incubated at 42 °C for 10 min. After centrifugation (3000× g) at 25 °C for 5 min, the supernatant was removed, and the pellet was resuspended in YPD. Transformants were selected by growth on synthetic complement minimal media lacking uracil (CSM-URA) at 30 °C for 2−3 days.

### 4.3. Microscopy

To observe yeast cells expressing Pma1p-EGFP, cells were grown in YPD at 30 °C overnight, washed with sterile distilled water, mounted on a pad of 2% low-melting agarose formed on a slide, and analyzed using a fluorescence microscope (Nikon Eclipse Ti-U, Nikon, Japan) equipped with a Nikon Plan Apo 100×, 1.45/NA oil immersion objective.

### 4.4. Measurement of RLS Using a Tetrad Dissection Microscope Equipped with a Micromanipulator

Yeast cells were grown in YPD at 30 °C overnight, diluted to an OD_600_ of 0.15, and further grown at 30 °C until OD_600_ reached 0.5−0.7. The cells were streaked along one side of a YPD agar plate and incubated at 30 °C for 2−3 h. Using a micromanipulation dissection microscope (MSM400, Singer Instruments, United Kingdom), a grid of reference holes was established on the growth surface of the YPD plate. Two or three small healthy yeast cells were transferred to each reference hole. From these cells, 1−3 virgin mother cells per hole were established by separating and discarding mother cells for the first two generations, with the aim of establishing 20−50 virgin mother cells per experiment. Daughter cells were separated and discarded immediately after they budded off. Plates were maintained at permissive temperature (30 °C) and stored at 4 °C overnight throughout the experiment.

### 4.5. Vacuole Staining with FM4/64

Vacuoles of growing yeast cells were labeled with FM4/64 as previously described with minor modifications (Vida and Emr, 1995 JCB). Briefly, BY4742, BY4742 *vac8*Δ, or BY4742 *vph1*Δ cells were grown at 30 °C to an OD600 of 0.5 in YPD. Then, the cells were incubated with aeration in YPD containing 1 mM FM4/64 (Molecular Probes, USA) at 30 °C for 1 h. After the cells were harvested by centrifugation (2400× *g*) at room temperature for 1 min, washed with fresh YPD 3 times, and further grown in YPD at 30 ℃ for 2 h, vacuoles were analyzed by a fluorescence microscope (Nikon Eclipse Ti-U) equipped with a Nikon Plan Apo 100×, 1.45/NA oil immersion objective.

### 4.6. Protein Interaction Network Construction and Proximity Analysis

Based on the protein interactome of BioGRID, we selected interactions with yeast origins (NCBI taxonomy ID: 559292). Based on the walk-trap functions of the R-igraph package, we identified protein clusters and their interactions. These were visualized as a network using the Cytoscape program. To calculate network proximity, we checked binding partners of protein pairs (i.e., protein A and B) and calculated the degree of sharing partners based on Jaccard scores. After calculation of the network proximity score, we normalized the 12 genes in a heatmap using Z-score transformation for comparison. Lists of 57 CLS- and 238 RLS-associated genes were obtained from previous studies (Table S4 in Reference [28] and Table S2 in Reference [35]). Controls were the scores normalized to any background genes in the network. For the in-depth investigation, we generated PPI subnetworks from the protein interactome of BioGRID by choosing direct interaction partners of 57 CLS genes found in a previous study [28] and the 12 genes regulating Pma1p asymmetry (Table 1).

## 5. Conclusions

Our work identified a subset of endocytic genes as key modulators of Pma1p asymmetry during cell division in Saccharomyces cerevisiae and suggests that Pma1p asymmetry, *per se*, is not a major determinant of the aging of mother cells and the rejuvenation of daughter cells.

## Figures and Tables

**Table 1:** List of deletion strains isolated from a genome-wide screen in which the asymmetric distribution of Pma1p-EGFP during cell division was disrupted.

Strain	Percentage of Cells with the Class II Phenotype	Gene Name	Known Functions
Wild-type BY4742	30		
BY4742 *vps9*Δ	89	*VPS9*	A guanine nucleotide-exchange factor (GEF) that functions in vacuolar protein transport by stimulating Vps21p
BY4742 *vps8*Δ	88	*VPS8*	A component of the CORVET complex involved in endosomal vesicle tethering and fusion in the endosome-to-vacuole protein targeting pathway
BY4742 *vps21*Δ	76	*VPS21*	An endosomal Rab family GTPase required for endocytic transport and sorting of vacuolar hydrolases and endosomal localization of the CORVET complex
BY4742 *vam7*Δ	72	*VAM7*	A vacuolar SNARE protein that functions with Vam3p and Vti1p in vacuolar protein trafficking
BY4742 *vps3*Δ	71	*VPS3*	A component of the CORVET membrane tethering complex
BY4742 *vps17*Δ	65	*VPS17*	A subunit of the retromer complex essential for endosome-to-Golgi retrograde transport
BY4742 *vps26*Δ	62	*VPS26*	A vacuolar component of the retromer complex
BY4742 *vps29*Δ	58	*VPS29*	A subunit of the retromer complex essential for endosome-to-Golgi retrograde transport
BY4742 *vps41*Δ	56	*VPS41*	A subunit of the HOPS endocytic tethering complex involved in vacuolar membrane fusion
BY4742 *mon1*Δ	53	*MON1*	A subunit of the heterodimeric Mon1p-Ccz1p GEF complex that stimulates activation of Ypt7p
BY4742 *vps35*Δ	51	*VPS35*	An endosomal subunit of the retromer complex required for retrograde transport
BY4742 *ypt7*Δ	50	*YPT7*	A Rab family GTPase required for vacuole fusion

100~300 cells per wild-type or each mutant were analyzed.

## Data Availability

The data presented in this study are contained within the article.
